# Effects of robot-assisted versus hand-assisted nephroureterectomy on circulating tumor cells for upper urinary tract urothelial carcinoma

**DOI:** 10.1038/s41598-021-99092-4

**Published:** 2021-09-30

**Authors:** Hui-Kung Ting, Tai-Lung Cha, Yi-Ta Tsai, Shu-Yu Liu, Sheng-Tang Wu, En Meng, Chih-Wei Tsao, Chien-Chang Kao, Chin-Li Chen, Guang-Huan Sun, Dah-Shyong Yu, Ming-Hsin Yang

**Affiliations:** 1 Division of Urology, Department of Surgery, Tri-Service General Hospital, National Defense Medical Center, No. 325, Cheng-Kung Rd, Sec 2, Neihu 114, Taipei, Taiwan; 2 Graduate School of Medical Sciences, National Defense Medical Center, Taipei, Taiwan

**Keywords:** Surgical oncology, Cancer, Urology

## Abstract

To compare perioperative circulating tumor cells (CTC) in primary upper tract urothelial carcinoma (UTUC) patients who underwent hand-assisted retroperitoneoscopic nephroureterectomy (HANU) or robotic-assisted nephroureterectomy (RANU). A total of 29 patients received RANU (n = 10) or HANU (n = 19). Peripheral blood samples were collected before, 24 h after surgery (POh24) and on postoperative day 28 (POD28). The demographic and pathologic data are similar in both groups. RANU had a longer operative time (*p* = 0.031), less bleeding volume (*p* = 0.004), and comparable pain sore (*p* = 0.169). The mean CTC numbers before surgery (2.4 vs. 2.3, *p* = 0.482), POh24 (2.4 vs. 1.9, *p* = 0.668) and POD28 (0.5 vs. 0.6, *p* = 0.280) were not significant different among groups. The amount of CTCs in both groups decreased and reached similar level on POD28. No significant difference of overall and intravesical recurrence rate between the two approaches. In comparison to RANU, more surgical manipulation does not affect tumor cell translocation into the bloodstream in UTUC patients who received HANU. However, a longer follow-up would be needed for the final comparison of tumor recurrence.

## Introduction

Upper-tract urothelial carcinoma (UTUC) indicated urothelial tumors from renal calyces to distal ureter, which account for 5–7% of all renal tumors and 5–10% of all urothelial cancers^1^. Standard treatment for high-risk UTUC is open radical nephroureterectomy (ONU) with excision of bladder cuff, and growing evidence suggested that equivalent oncological outcome by minimally invasive approach (laparoscopic or robotic)^2^. On the other hand, most studies showed hand-assisted retroperitoneoscopic nephroureterectomy (HANU) had comparable oncological outcomes when compared with ONU^3,4^. However, report on comparison between robotic-assisted laparoscopic nephroureterectomy (RANU) and HANU is scarce.

Both animal and clinical studies have showed that circulating tumor cells (CTC) can be shed into the bloodstream during surgery and soft manipulation is able to reduce the effect^5,6^. To our knowledge, perioperative detection of CTCs for UTUC patients who undergone nephroureterectomy has not been reported. In comparison to RANU, HANU had much more manipulation to the tumor and theoretically would cause more tumor cell translocation into the circulation.

In our study, we investigated whether nephroureterectomy promotes the release of CTCs in primary UTUC patients and exam the hypothesis if HANU may cause more CTCs in comparison to RANU.

## Methods

### Patients and blood sample collection

All patients signed their written informed consent. We conducted this study in accordance with the guidelines stipulated in the Helsinki Declaration. The study was approved by the Ethical Committee of Tri-Service General Hospital, Taiwan (TSGHIRB NO: 2-107-05-167). The study includes 29 UTUC patients who had no previous bladder cancer history and received RANU (n = 10) or HANU (n = 19) by six different urologists at Tri-Service General Hospital. Peripheral blood samples were collected before surgery, POh24 and POD28. 7.5 mL of blood were collected in EDTA tubes in each patient. All samples were processed within 4 h after collection and then further evaluated for CTC analysis.

### Immunomagnetic beads preparation for CTC isolation

The original IsoFlux™ CTC enrichment protocol was modified to enable the replacement of IsoFlux™ beads with CELLection™ Dynabeads (Invitrogen). CELLection™ Dynabeads® coated with a human anti-mouse IgG (Invitrogen) were utilized for enrichment of the cells. Dynabeads were incubated with EPCAM (Ber-EP4, Abcam) antibody (0.02 µg antibody/µl bead suspension) at room temperature. After incubation, washed the beads with PBS with 0.1% bovine serum albumin (BSA) twice and stored the beads at 4 °C.

### Sample preparation

Red blood cells (RBC) were lysed in 45 mL Red Blood Cell lysis buffer containing 0.01 M potassium hydrogen carbonate, 0.155 M ammonium chloride, and 0.1 mM EDTA. After centrifugation, the cell pellet was washed with PBS. The cells were resuspended in RPMI medium containing 1% FBS, 1 mM CaCl_2,_ and 5 mM MgCl_2_ followed by adding the antibody-coated beads according to the original blood volume. Cells were then incubated with magnetic beads at 4 °C.

### IsoFlux™ and CTC enrichment

IsoFlux™ (Fluxion Biosciences Inc, USA) utilizes immunomagnetic beads targeted to antigens expressed on the cell surface. The beads are magnetic cores surrounded by polymeric layer coated monoclonal human anti-mouse IgG antibody. In combination with primary mouse IgG antibodies allows enrichment of CTCs in the microfluidic device. The sample is positively isolated from the sample while the cells flow through a microfluidic cartridge designed for cell isolation.

### Sample isolation and collection

Beads holding the cancer cells were retrieved by running the enrichment protocol on the IsoFlux™ machine (Fluxion). Isolated cells were then recovered in 200 µl RPMI medium containing 1% FBS, 1 mM CaCl_2,_ and 5 mM MgCl_2_ followed by transferring to a low retention microcentrifuge tube (Fisher). For enabling removal of supernatant, a cylinder magnet was used to pull down cells bound to beads towards the bottom of the tube. Cells were then fixed in 4% PFA and added onto glass slides, on which a circle with the same size as the magnet had been drawn using a water repellent Dako pen. The glass slide was placed on top of the magnet when adding or removing buffer from cells.

### CTC analysis and immunofluorescence

Isolated cells were mounted and fixed on slides and were blocked for 5 min in 10% normal donkey serum (NDS). Hereafter cells were then stained with PE-conjugated anti-CD45 [5B-1] antibody (1:200; MACS Miltenyi Biotec). Cells were then permeabilized using 0.2% Triton X-100 in PBS containing 0.5% BSA and 2 mM EDTA followed by staining of the cells with FITC-conjugated anti-Cytokeratin [CK3-6H5] antibody (1:10; MACS Miltenyi Biotec). To stain the cell nuclei, cells were incubated in DAPI afterwards. The sample was mounted with Dako Faramount Aqueous Mounting Medium. Images were captured with a fluorescent microscope (Axio Scan.Z1, Zeiss). CTCs are defined as CK-positive, CD45-negative, and nucleated cells.

### Statistical analysis

In general, all experiments were performed at least 3 independent times, unless stated. Propotional hazards model, performed with SPSS (SPSS v25.0, IBM Corp, USA) was used for recurrence survival analysis. Multivariable regression analysis, also analyxed by SPSS, addressed CTC changes before and after surgeries. Unless stated otherwise, results for continuous variables are presented as mean ± S.D. Statistically significance was determined using the Mann–Whitney test. Data were analyzed using Prism version 9 (GraphPad Software Inc, USA). Two-tailed *P* values of less than 0.05 was considered significant for all tests.

## Results

### Patient characteristics

Our cohort included 10 patients in the RANU group and 19 patients in the HANU group. Detailed patient characteristics are shown in Table 1. There was no significant difference in age, gender, body mass index, tumor location, or hydronephrosis among the 2 groups. Further analysis showed no significant difference in tumor grade, pathological T-stage, N-stage (pN), presence of concomitant carcinoma in situ (CIS), lymphovascular invasion and adjuvant chemotherapy. The mean age was 62.5 ± 6.9 years in the RANU group and 63.2 ± 9.2 years in the HANU group (*p* = 0.830). In both groups, the patients compassed a slight female predominance (60%), most tumors are kidney involved (> 60%), at least stage II (around 60%), and high grade (> 70%). No history of prior bladder cancer, cystectomy, or concurrent bladder tumor in both groups were found.

**Table 1:** Characteristics of UTUC Patients.

	RANU n = 10	HANU n = 19	*p* value
Age (mean ± SD)	62.5 ± 6.9	63.2 ± 9.2	0.830
Gender			
Male	4	7	> 0.999
Female	6	12	
BMI (mean ± SD)	26.2 ± 3.0	25.6 ± 2.7	0.580
Tumor location			
Kidney	5	10	0.770
Ureter	3	7	
Both	2	2	
T stage			
1	4	8	0.774
2	4	9	
3	2	2	
Grade			
High	8	14	> 0.999
Low	2	5	
Hydronephrosis			
Yes	7	11	0.694
No	3	8	
pN			
pN+	2	2	0.592
pN0	8	17	
Lymphovascular invasion			
Yes	3	3	0.633
No	7	16	
Hx of bladder cancer			
Yes	1	9	> 0.999
No	9	17	
Concomitant CIS			
Yes	1	9	> 0.999
No	9	17	
Adjuvant chemotherapy			
Yes	3	4	0.665
No	7	15	

*p < 0.05 indicates statistical significance.

### Perioperative and oncological outcome of RANU and HANU

Table 2 provides the details of the perioperative outcomes and oncological outcomes. In comparison to HANU, RAUN had a longer mean operative time (240 ± 37.3 min vs. 209.7 ± 47.5 min, *p* = 0.028), less bleeding volume (120 ± 137.8 mL vs. 347.4 ± 356.9 mL, *p* = 0.002), and similar pain score (6.2 ± 1.5 vs. 5.4 ± 2.0, *p* = 0.168).

**Table 2:** Perioperative and Oncological Outcome of UTUC Patients.

Variable (mean ± SD, range)	RANU n = 10	HANU n = 19	*p* value
Surgery Duration, hours	240 ± 37.3 (176–300)	209.7 ± 47.5 (157–343)	0.028*
Bleeding volume, mL	120 ± 137.8 (50–500)	347.4 ± 356.9 (50–1500)	0.002*
Pain score	6.2 ± 1.5 (4–9)	5.4 ± 2.0 (3–10)	0.168
Median follow up, months	12.9 ± 3.4 (9–20)	17.5 ± 2.5 (14–22)	0.001*
Positive surgical margin	0	0	> 0.999
Bladder recurrence	1 (10%)	3 (16%)	> 0.999
Cancer recurrence (other than bladder)	1 (10%)	2 (11%)	> 0.999
Cytology before surgery	4 (40%)	5 (26%)	0.675
Cytology after surgery	2 (20%)	2 (11%)	0.592

*p < 0.05 indicates statistical significance.

The median follow-up time of the RANU group and the HANU group was 12.9 months and 17.5 months, respectively (*p* = 0.001). No subjects in both group had positive surgical margin. During follow up period, one case (10%) in the RANU group and three cases (16%) in the HANU group were proven to have bladder recurrence (*p* > 0.999). Non-bladder recurrence was observed in one of 10 (10%) RANU patients and two of 19 (11%) HANU patients (*p* > 0.999). On propotional hazards model analyses, surgery type was also not significantly associated with intravesical recurrence (*p* = 0.864; Hazard ratio 1.25; 95% CI 0.1–15.5). One case of tumor recurrence in previous renal fossa was noted in the HANU group (stage: T2N0, high grade; tumor location: kidney, no hydronephrosis).

For each patient, urine samples were collected before surgery (by self-void) and immediately after surgery (from urine bag). The positive rate of urine cytology before surgery (40% vs. 26%) and after surgery (20% vs. 11%) were no different among the RANU group and HANU group (*p* = 0.675 and *p* = 0.592, resp.).

### Detection of circulating UTUC cells

**Figure 1:**
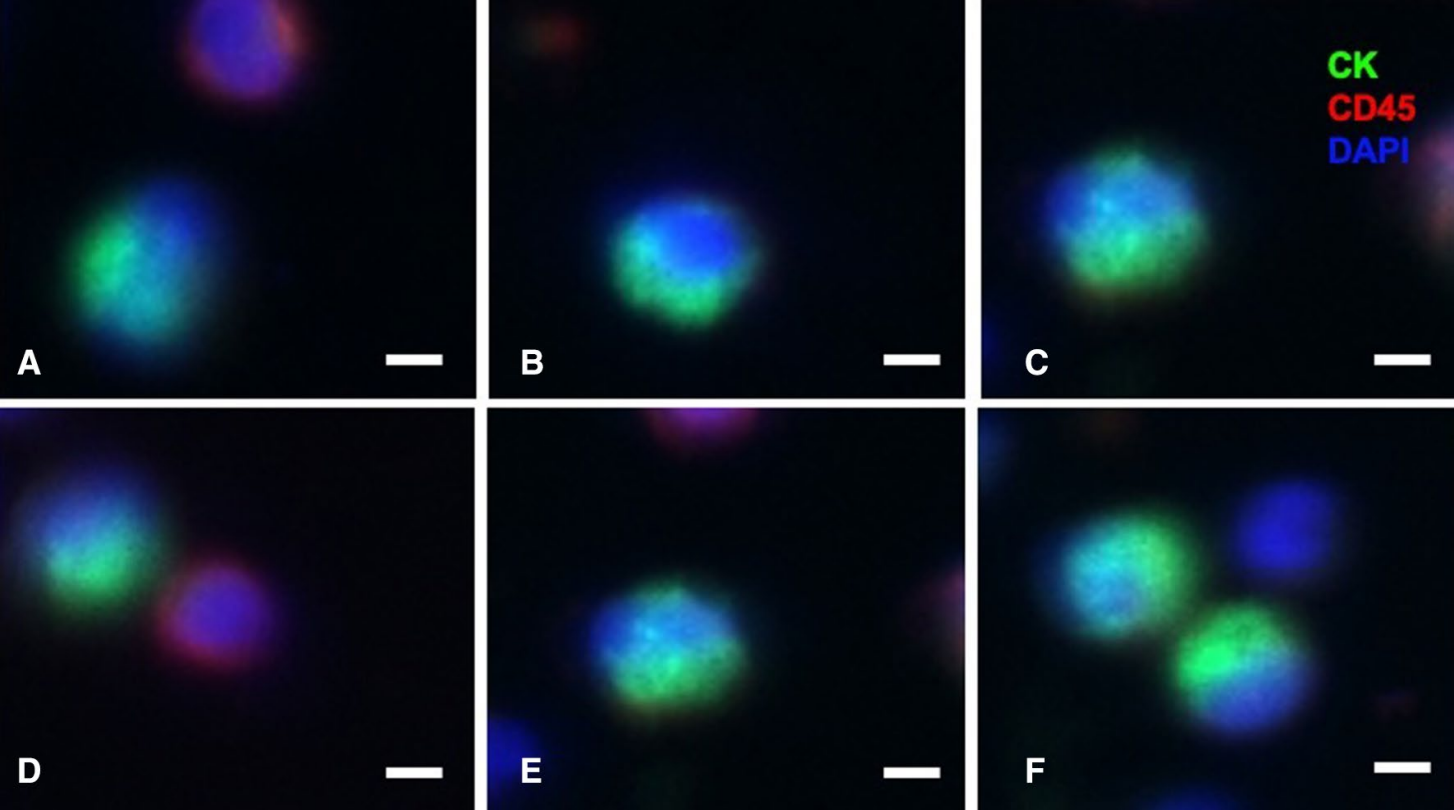
Circulating tumor cells are present in blood samples from patients. (**A**–**F**) Immunofluorescence staining of representative cells obtained from IsoFlux. Cancer cells fulfilled criteria for CTCs, including: nucleated (blue), CK-positive (green), and CD45-negative cells (non-red). Scale bar, 10 µm.

**Figure 2:**
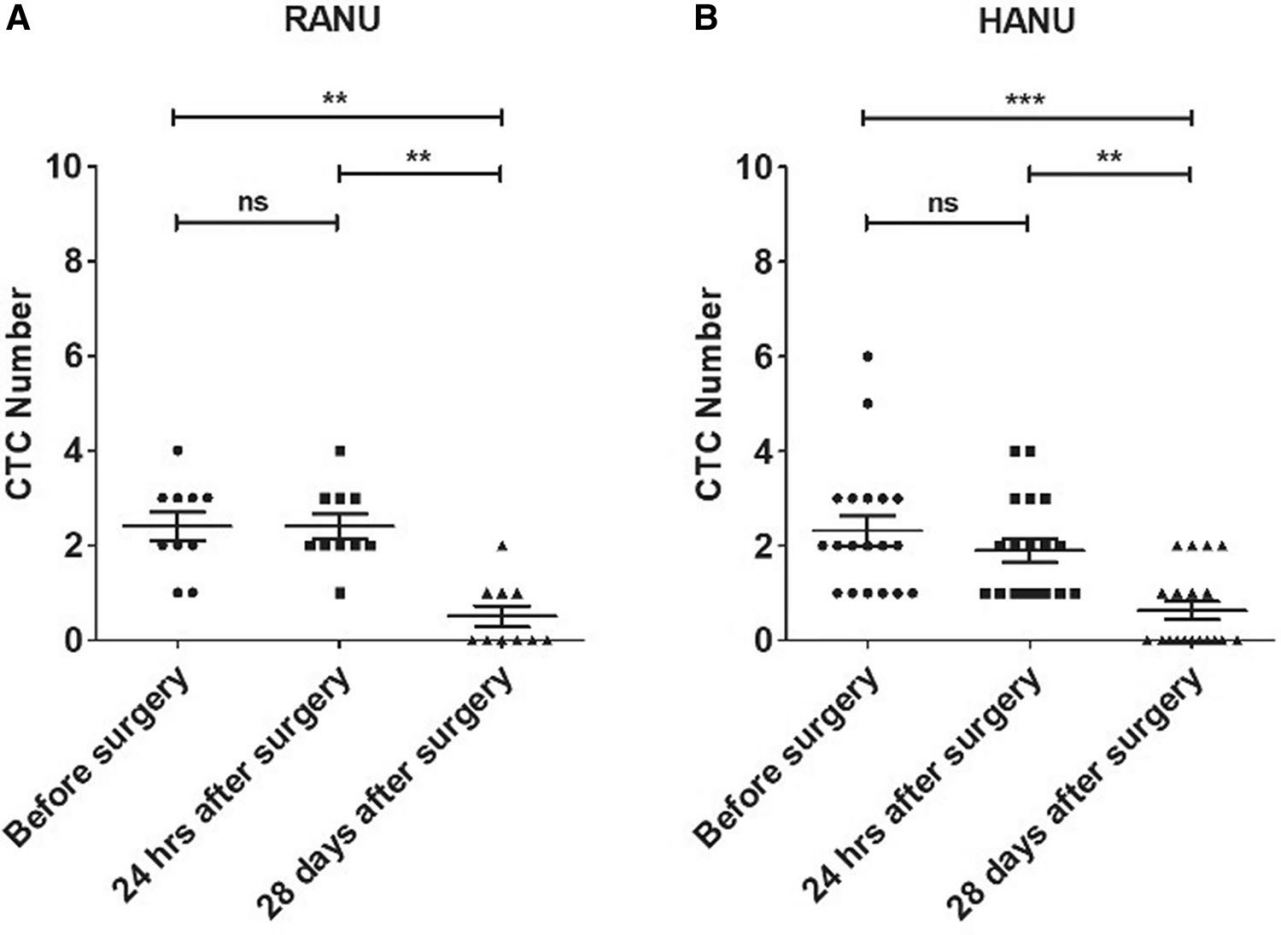
Time course analysis of CTC levels in UTUC. CTC levels were evaluated in before surgery and at 24 h and 28 days after surgery. Numbers of CTCs/7.5 ml are showed on the plots; (**A**) Results from patients who received RANU; (**B**) Results from patients who received HALU; Mann–Whitney test; **p < 0.01; ***p < 0.001.

Peripheral blood samples were drawn before, 24 h after surgery (POh24) and on postoperative day 28 (POD28). CTCs were enriched and detected in all patients affected by UTUC after immunofluorescence (IF) staining of cells. CTCs are defined as CK-positive, CD45-negative, and nucleated cells (Fig. 1A–F). The CTC numbers (mean CTCs/7.5 mL) in the patients before and at different time-points after surgery are shown in Table 2. We are able to detect CTCs from all of the patients in our cohort. Compare with the preoperative CTC counts, there was no difference in postoperative CTC counts at POh24 (*p* = 0.276) but it decreased significantly at POD28 (*p* < 0.0001) in the whole cohort. The same finding was also noted in different surgical techniques (Fig. 2).

### Effect of different surgeries and perioperative outcomes on CTCs

**Figure 3:**
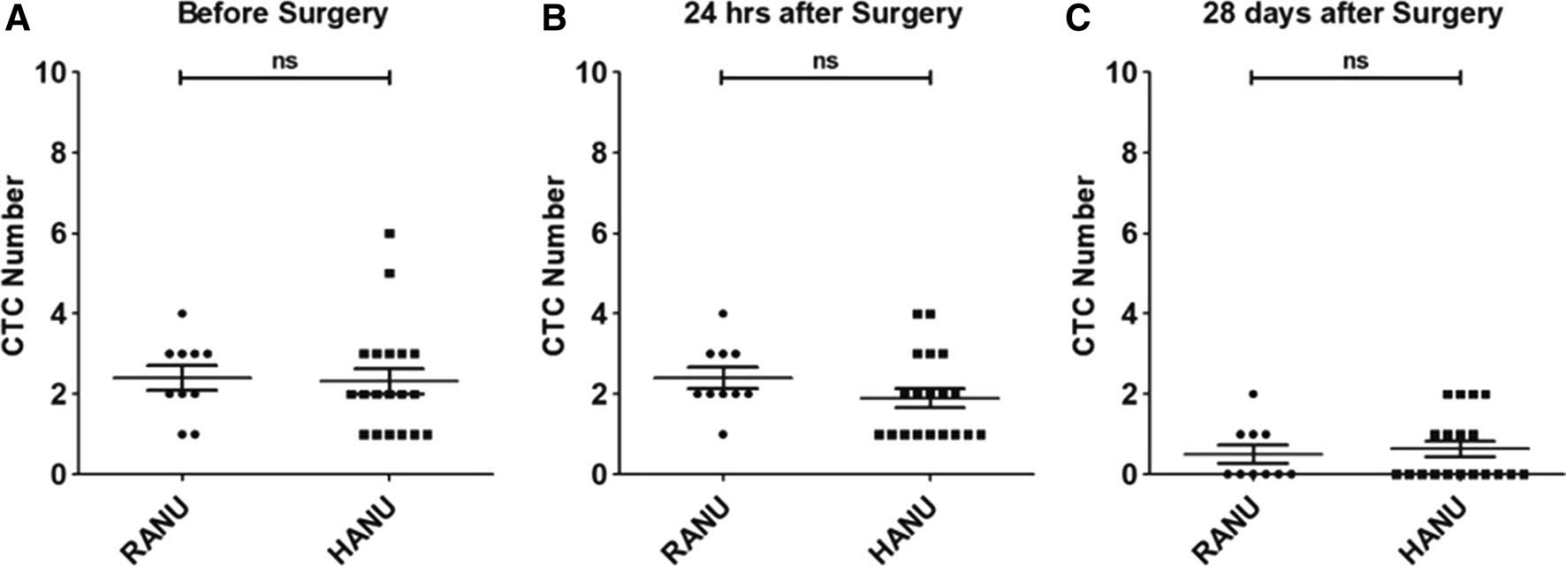
Correlation of CTC counts with different surgical techniques at various time points; (**A**) CTC counts before surgery; (**B**) CTC counts 24 h after surgery; (**C**) CTC counts 28 days after surgery; ns, not significant.

Correlation of CTC counts with different surgical techniques at various time points are shown in Fig. 3. The mean CTC numbers before surgery (2.4 ± 1.0 vs. 2.3 ± 1.4), POh24 (2.4 ± 0.8 vs. 1.9 ± 1.0) and POD28 (0.5 ± 0.7 vs. 0.6 ± 0.8) were not significant different between the RANU group and HANU group (*p* = 0.482, *p* = 0.668 and *p* = 0.280, resp.). On multivariable analyses, none of the perioperative outcomes were independent predictors of the change of CTCs before and after surgeries (Supplementary table 1).

## Discussion

Our patient characteristics (60% at least stage II, 70% high grade) are similar to the previous report which mentioned that Asian upper tract urothelial carcinoma (UTUC) patients are likely to have a more advanced stage and higher-grade disease at presentation^1^. In contrary to the previous data, there is slightly more female patients (60%) in our cohort, which may be related to the small patient numbers.

There are few studies that describe direct comparisons between RANU and HANU. Hu. et al. conducted a matched comparison of RANU (N = 18) and HANU (N = 18) which showed RANU have a similar operative time (255.17 vs. 250.17 min, *p* = 0.333) but significantly less blood loss (68.89 vs. 358.33 mL, *p* < 0.001) and higher pain score (6.22 vs. 3.93, *p* = 0.043)^7^. In our cohort, the RANU group also had significantly less blood loss (120 vs. 347.4 mL, *p* = 0.002), but the operative time is longer (240 vs. 209.7 min, *p* = 0.028) and the pain score was not significantly different (6.2 vs. 5.4, *p* = 0.168) in comparison with the HANU group. The longer operative time but less surgical blood loss in the RANU group may be due to re-docking of the robotic machine and a more meticulous dissection during the surgery. Similar pain scores between the two groups might be relevant to the major wound length and the location of abdominal wall (for both specimen extraction and bladder cuff excision) are almost identical.

In our study, the overall tumor recurrence (20% vs. 26%, *p* > 0.999) and intravesical recurrence (IVR, 10% vs. 16%, *p* > 0.999) did not significantly differ between the RANU and the HANU groups which was consistent with previous report by Hu et al. (IVR, 11.1% vs. 33.3%, *p* = 0.849). However, a meta-analysis showed the median time to IVR was 22.2 months (6.7–56.5)^8^. Our median follow-up time is only 12.9 months in RANU and 17.5 months in HANU patients, a longer follow up is necessary to determine whether there are any differences in the two surgical approaches in regard of IVR.

Manipulation during the surgical procedure raises the concern of tumor cell translocation into circulation. A number of reports showed intraoperative tumor cell shedding in different cancer types including sarcomas, hepatocellular, breast, renal, and prostate origin^9^. For urothelial carcinoma, it has also been proved that transurethral resection of bladder cancer can release tumor cells into the bloodstream^10^. Previous studies have reported that HANU is an independent risk factor of recurrence and has significantly higher overall and intravesical recurrence rates compares to other approaches^11,12^. HANU has much more vigorous manipulation in comparison to RANU and theoretically may have a higher chance of CTC shedding. To our knowledge, perioperative detection of CTCs for UTUC patients who undergone nephroureterectomy has not been reported. In our study, POh24 CTCs number showed no significant change in both groups (Fig. 2) nor difference among the two groups (Table 2). This finding indicates that the above presumption of hand-assist surgery might have higher POh24 CTC than robotic approach is not true. Other indirect evidence includes no significant difference in the rate of positive postoperative urine cytology nor recurrence (Table 2). Furthermore, POD28 CTC numbers in both groups showed a significant decline and without difference between two different surgical approaches (Figs. 2, 3). The decrease of POD28 CTC numbers demonstrates that surgical removal of the primary tumor by either HANU or RANU can effectively decrease CTC.

Main limitation of our study is that the relatively small number of patients. Both surgeons’ and patients’ preference determine the operation methods which may lead to bias. Furthermore, relative short follow up time failed to show any relationship between CTCs and tumor recurrence. Moreover, interpretation of our results should be careful because our sample are collected from peripheral blood. A substantial difference in the number and positive rate of CTC at different vascular locations had been reported in several types of cancer^13–15^. The peripheral blood had been filtered by the pulmonary microvascular system and mixed with venous blood from other body parts which might underestimate CTC numbers.

## Conclusions

Our data provide evidence that in comparison to RANU, more surgical manipulation does not affect tumor cell translocation into bloodstream in UTUC patients who received HANU. But a longer follow-up would be needed for final comparison of tumor recurrence.

## Supplementary Information

Supplementary Information.

## Data Availability

The dataset analyzed for the current study is available from the corresponding author on reasonable request.
