# Supplementary material for: Effects of robot-assisted versus hand-assisted nephroureterectomy on circulating tumor cells for upper urinary tract urothelial carcinoma
Source: Sci Rep. 2021 Sep 30;11:19499. doi: 10.1038/s41598-021-99092-4 (PMC8484450; doi:10.1038/s41598-021-99092-4)
Supplement: Supplementary file 1 — Supplementary Information. [file 41598_2021_99092_MOESM1_ESM.docx]

**Supplementary table 1. Correlation of Perioperative Outcome and CTC Changes**

| **Variable** | **POh24 (*p* value)** | **POD28 (*p* value)** |
| --- | --- | --- |
| **Age** | **0.537** | **0.417** |
| **Gender** | **0.858** | **0.344** |
| **BMI** | **0.896** | **0.401** |
| **Surgery Type** | **0.211** | **0.404** |
| **T stage** | **0.773** | **0.706** |
| **Tumor grade** | **0.413** | **0.130** |
| **Tumor location** | **0.155** | **0.551** |
| **Hydronephrosis** | **0.132** | **0.506** |
| **N stage** | **0.137** | **0.652** |
| **Lymphovascular invasion** | **0.872** | **0.572** |
| **History of bladder cancer** | **0.243** | **0.999** |
| **Concomitant CIS** | **0.370** | **0.720** |
| **Cytology before surgery** | **0.384** | **0.373** |
| **Cytology after surgery** | **0.366** | **0.993** |
| **Surgery duration, hours** | **0.315** | **0.587** |
| **Bleeding volume** | **0.759** | **0.120** |
| **Bladder recurrence** | **0.565** | **0.470** |
| **p* < 0.05 indicates statistical significance. | | |
